# Linear and nonlinear analyses of the association between low–density lipoprotein cholesterol and diabetes: The spurious U–curve in observational study

**DOI:** 10.3389/fendo.2022.1009095

**Published:** 2022-11-17

**Authors:** Yujia Ma, Zechen Zhou, Xiaoyi Li, Kexin Ding, Han Xiao, Yiqun Wu, Tao Wu, Dafang Chen

**Affiliations:** Department of Epidemiology and Biostatistics, School of Public Health, Peking University, Beijing, China

**Keywords:** diabetes, low-density lipoprotein cholesterol (LDL-C), fasting blood glucose (FBG), linear causal association, cardiometabolic disease

## Abstract

**Objective:**

Hyperlipidemia is traditionally considered a risk factor for diabetes. The effect of low-density lipoprotein cholesterol (LDL-C) is counterintuitive to diabetes. We sought to investigate the relationship between LDL-C and diabetes for better lipid management.

**Methods:**

We tested the shape of association between LDL-C and diabetes and created polygenic risk scores of LDL-C and generated linear Mendelian randomization (MR) estimates for the effect of LDL-C and diabetes. We evaluated for nonlinearity in the observational and genetic relationship between LDL-C and diabetes.

**Results:**

Traditional observational analysis suggested a complex non-linear association between LDL-C and diabetes while nonlinear MR analyses found no evidence for a non-linear association. Under the assumption of linear association, we found a consistently protective effect of LDL-C against diabetes among the females without lipid-lowering drugs use. The ORs were 0.84 (95% CI, 0.72–0.97, *P=0.0168*) in an observational analysis which was more prominent in MR analysis and suggested increasing the overall distribution of LDL-C in females led to an overall decrease in the risk of diabetes (*P=0.0258*).

**Conclusions:**

We verified the liner protective effect of LDL-C against diabetes among the females without lipid-lowering drug use. Non-linear associations between LDL-C against diabetes in observational analysis are not causal.

## 1 Introduction

Diabetes is a complex metabolic disease regulated by a combination of environmental and genetic factors. Individuals with metabolic syndrome (MetS) have significantly increased risk for diabetes, independent of many other risk factors (1). Metabolic dyslipidemia, another main component of metabolic syndrome, is intricately associated with the development of diabetes. There seems to be a vicious circle between the onset of diabetes and dyslipidemia. Diabetic dyslipidemia is typically characterized by elevated serum triglycerides and low high-density lipoprotein cholesterol (HDL-C) concentrations, together with raised apolipoprotein B and the prevalence of smaller, denser LDL-C particles (2, 3).

Although the cluster of lipid abnormalities associated with type 2 diabetes is defined by a high concentration of TG and small dense LDL and a low concentration of HDL cholesterol (4, 5), the effect of LDL-C has been controversial. The circulating level of LDL-C is considered as a causal life-cycle risk factor for cardiovascular disease, while the effects of LDL appear to be heterogeneous in the context of shared risk factors. It’s of great importance to decipher the relationship between LDL-C and diabetes especially when cardiometabolic multimorbidity, one of the most replicable multimorbidity profiles, has been a global health challenge (5–7). Several lines of evidence suggest that decreased levels of circulating LDL-C are associated with increased diabetes risk. Individuals with low levels of circulating LDL-C (e.g.,<60 mg/dL) exhibit a higher risk of prevalent and incident diabetes, and among individuals with coronary disease, LDL-C and diabetes are inversely related (8–10). Lotta et al. reported people naturally randomized to lower LDL-C had a higher risk of type 2 diabetes compared with the reference group, which is consistent with previous studies (11, 12). Klimentidis et al. demonstrated phenotypic and genetic characterization of lower LDL-C and increased diabetes risk in the UK Biobank. They found levels of circulating LDL-C were negatively associated with diabetes prevalence (odds ratio 0.41 [95% CI 0.39, 0.43] per mmol/L unit of LDL-C) (13). White et al. have shown that genetically predicted that a 1-SD genetically instrumented elevation in LDL-C levels (equivalent to 38 mg/dL) is associated with a lower risk of type 2 diabetes (odds ratios (ORs) 0.79, 95% CI, 0.71-0.88) (14).

Existing researches, however, suggest that the inversed association between LDL-C and diabetes is largely mediated by lipid-lowering mediation. The systematic review derived from thirty-three randomized controlled trials found that there was no independent association between reduction in LDL cholesterol and incident diabetes while more intensive lipid-lowering therapy was associated with a higher risk of incident diabetes compared with less intensive therapy (risk ratio: 1.07; 95% CI, 1.03-1.11; *P<0.001*; I^2^ = 0%) (15). It’s still well-accepted that cardiovascular benefit from lipid-lowering therapy outweighs the increased risk for new-onset diabetes mellitus and clinical practice in patients with multiple cardiovascular risk factors or existing cardiovascular disease should not be modified (16, 17). However, the independent effects of LDL-C on diabetes should be carefully assessed for better diabetes management.

Another reason for the controversial association of LDL-C with diabetes is the impracticability of excluding confounding factors and reverse causality from the observational study. Mendelian randomization (MR) analysis has the advantage to circumvent this problem to some extent, which uses germline genetic variants as an instrumental variable (IV) to proxy for environmentally modifiable exposures within observational epidemiological studies and is considered analogous to RCT due to the law of independent assortment (18).

Therefore, in this study, we investigated both liner and non-liner associations between LDL-C and diabetes in observational and MR studies respectively to provide scientific basis for lipid management of diabetic patients.

## 2 Materials and methods

### 2.1 Subjects

The present study relied on the Fangshan Family-based Ischemic Stroke Study in China (FISSIC) (19). FISSIC is an ongoing community-based case-control genetic epidemiological study that started in June 2005 in Fangshan District, a rural area located southwest of Beijing, China. We used the baseline data for the second phase of the FISSIC study. 9540 participants aged ≥40 years were recruited either by responding to recruitment posters about the study or by invited phone calls if they had health medical records in community health centers between December 2011 and April 2012 (20).

This study was approved by the Ethics Committee of the Peking University Health Science Center (Approval number: IRB00001052-13027), and written informed consent was provided by all participants.

### 2.2 Data collection

In the FISSIC study, baseline data including sociodemographic status, education, occupation, diet, lifestyle, health behavior, and medical history, of all participants were collected through a face-to-face questionnaire survey by trained staff. For smoking, we assigned current smokers and former smokers as ‘ever smokers’ to avoid a misleadingly elevated risk for the reference group. Body mass index (BMI) was calculated as kg/m2. Seated position BP was measured after resting for at least 5 min. The systolic BP (SBP) and diastolic BP (DBP) used in the analysis were calculated as the mean of three consecutive measurements for each participant. Hypertension was defined as self-reported history of hypertension or SBP≥140 mmHg or DBP≥90 mmHg or use of antihypertensive medications. After overnight fasting of at least 12 hours, a venous blood sample was obtained from the forearm of each participant. Serum or plasma samples were separated within 30 minutes of collection and were stored at -80 °C, which were used for measurement of fasting blood glucose and the standard 75-g oral glucose tolerance test, total cholesterol, low-density lipoprotein cholesterol, high-density lipoprotein cholesterol, triglycerides, creatinine concentrations, and DNA analysis. Laboratory tests of serum lipid levels, including TG, TC, HDL-C, and LDL-C, were performed by qualified technicians from the Laboratory of Molecular Epidemiology in the Department of Epidemiology at Peking University. Serum glucose, blood urea nitrogen, creatinine, concentrations of total cholesterol, triglycerides, low-density lipoprotein cholesterol, and high-density lipoprotein cholesterol were measured by using the Hitachi 7180 autoanalyzer (Hitachi High-Technologies Corp., Tokyo, Japan) (19). Individuals with extreme outlier values (outside the range of Q3 +1.5×IQR and Q1-1.5×IQR) were recorded as missing values over concern that these recordings were likely to be inaccurate. Variables with missing rates of more than 10% were discarded and not included in the analysis. We used multiple imputation to impute variables with missing data, which was implemented using “mice” package in the R version 3.6.1.

### 2.3 Outcomes

Our primary outcome of interest was diabetes, which was defined if one of the following inclusions was met: 1) self-reported diabetes status; 2) hypoglycemic drugs use; 3) fasting blood glucose (FBG)≥7.0 mmol/L; 4) two hours blood after glucose oral glucose tolerance test (OGTT) ≥ 11.1 mmol/L. We took FBG as the secondary outcome to further assess the effect of LDL-C on diabetes. Individuals with missing values of the above variables were excluded.

### 2.4 Genotype

DNA was extracted using a LabTurbo 496-Standard System (TAIGEN Bioscience Corporation, Taiwan, China). In addition, the purity and concentration of DNA were measured using ultraviolet spectrophotometry. Furthermore, the genomic DNA sample was genotyped with Infinium Asian Screening Array (Agena Bioscience, San Diego, CA). We used two negatives (blanks) and three positive controls to control the quality of the genotyping process, and the results were satisfied. We also chose 5% samples randomly for repeat analysis to verify the reproducibility of the genotyping data. Plate-, individual-, and variant-level checks were conducted to exclude poor-quality genotype calls from the data set. The individual-based quality control criteria included a call rate of<99%, gender mismatch, excess heterozygosity, and relatedness. Variant-level quality control was performed to exclude variants with low cluster scores, low call rate (<99.9%), and those that deviated from Hardy–Weinberg equilibrium (*P<1 × 10^−4^*). The correlation between allele frequency of our samples and those of East Asian samples from the 1000 Genomes Project was examined and a high correlation (*r^2^ =*0.98) was observed.

### 2.5 Statistical analysis

#### 2.5.1 Observational association evaluations

We first assessed the relationship between LDL-C and diabetes using traditional statistical approaches. We applied a logistic regression model to assess the influence of LDL-C on diabetes and a linear regression model to assess the influence of LDL-C on FBG, after controlling for age, sex, BMI, diabetes family history, dyslipidemia, coronary heart disease (CHD), and blood pressure. For linear regression of FBG, we added the additional adjustment for hypoglycemic drug use.

We used restricted cubic splines (RCS) (21) to fit and visualize nonlinear relations. The number of nodes was determined when the Akaike information criterion (AIC) (22) value met the minimum.

#### 2.5.2 Instrumental variable

We generated the genetic risk score (GRS) of LDL-C to use as an instrumental variable in our MR analysis. The risk score for LDL-C in each of our genotyped participants is a weighted sum of the effect size estimates from the East Asian meta-analyses (23) and the genotype in each selected single nucleotide polymorphisms (SNPs).


$$GRS_{j}=\sum_{j=1}^{M}\beta_{j}G_{ij}$$

The data in our analyses for the instrumental variable was to our knowledge the largest genome-wide association study (GWAS) summary statistics for the genetics proxies of LDL-C of East Asian ancestry. Strict selection criteria were used to select qualified SNPs and construct GRS. We extracted only SNPs that pass the genome-wide significance threshold (*P< 5×10^-8^*) for association with LDL-C as candidate SNPs. Secondly, the SNPs were eliminated if they were in linkage disequilibrium (LD) based clumping at an LD threshold of *r^2^* <0.001 for all variants within a 1Mb window and a secondary clumping with a threshold *r^2^* <0.1 for all variants within the same chromosome using reference LD from the 1000 Genomes project. And the SNPs that were not available in our chip were not used in the analysis. Combined with the information of selected SNPs and weights, we specified the “sum” model in the software PRSice 2.3.5 (2021-09-20, https://github.com/choishingwan/PRSice) to complete the calculation of GRS (24). Additionally, we estimated statistical power for the MR studies using the online website (https://shiny.cnsgenomics.com/mRnd/) (25). The equations for estimating power was elaborated in Supplementary Data. And the comparison between the non-centrality parameter (NCP) based approach for power calculating and the simulation method using one genetic variant was demonstrated in the original literature (25).

#### 2.5.3 Linear MR

We performed a two-stage MR analysis to model the linear effect of LDL-C. In the first stage regression, we regressed LDL-C on the GRS *via* linear regression to generate an estimate of the effect of the GRS on the LDL-C, adjusting for sex, age and age2. We use the F statistic of regression at this stage to evaluate the validity of GRS. F statistic >10 was considered valid. The associations of the resulting estimated LDL-C values were examined using linear regression with the same adjustments in an observational analysis.

#### 2.5.4 Nonlinear MR

MR analyses to assess for potential nonlinear effects of LDL-C on the outcomes were performed with a fractional polynomial method (26). Briefly, this method first calculated instrument variable free LDL-C by taking the residuals of the regression of LDL-C on the GRS and divided the participants into centiles of the instrument variable free LDL-C. In each stratum, we calculated the linear MR estimates (localized average causal effect estimates, LACE) of the effect of LDL-C on outcomes separately with the same procedure described above in the linear MR analysis. Then we performed a meta-regression of the localized average causal effect estimates against the mean of the exposure in each stratum in a flexible semiparametric framework by using the derivative of fractional polynomial models of degrees 1 and 2. A trend test, which assesses for a linear trend among the localized average causal effect estimates, and a fractional polynomial test, which assesses whether a non-linear model fits the localized average causal effect estimates better than a linear model were reported for non-linearity. The slope of the relation at different values of LDL-C was more important than the differences that extrapolate across the whole range of the distribution. The slope of the graph was the average causal estimate at that value of LDL-C. A statistically significant causal estimate at a particular LDL-C value was evidenced not when the confidence interval for the odds ratio excludes the value 1, but when the slopes of the upper and lower bounds of its confidence interval are both positive for a positive estimate, or both negative for a negative estimate. This analysis was performed based on the *nlmr* R package (27).

The statistical analysis was performed from June 2021 to May 2022 using R statistical software version 3.6.0 (R Project for Statistical Computing).

## 3 Results

### 3.1 Demographics and baseline information

In aggregate, we included 4,876 individuals with sufficiently reliable baseline information and genotypes to evaluate the relationship between LDL-C with the risk of diabetes (**Supplementary Figure 1**). Summary information of the baseline characteristics of the participants is provided in **Table 1** and **Supplementary Tables**. There were 1180 participants defined as diabetes cases, with a prevalence of 24.2%. Among them, 57.8% (n=682) reported diabetes history; 52.8% (n=623) reported hypoglycemic drugs use. All cases had a FBG≥7.0 mmol/L and OGTT ≥ 11.1 mmol/L. At baseline, the diabetes group has a higher proportion of males (*P<0.0001*), obese (*P<0.0001*), former smokers (*P=0.0004*), have a diabetes family history (*P<0.0001*), have CHD (*P*<0.0001) and high blood pressure (*P<0.0001*), and use lipid-lowering drugs (*P<0.0001*). The levels of TG, HDL, and SBP were consistent with expectations. No significant difference was found in TC, LDL-C, and DBP.

**Table 1:** Summary demographic information of participants in observational analysis.

Characteristics		Overall	Control group	Diabetes group	*p*
No of participants		4876	3696	1180	
Sex (%)	Male	1814 (37.2)	1289 (34.9)	525 (44.5)	<0.0001
	Female	3062 (62.8)	2407 (65.1)	655 (55.5)	
Age at baseline (median [IQR], years)		56.00 [51.00, 62.00]	55.00 [50.00, 60.00]	59.00 [54.00, 67.00]	<0.0001
BMI (median [IQR], kg/m^2^)		25.82 [23.73, 28.01]	25.63 [23.59, 27.77]	26.51 [24.40, 28.56]	<0.0001
BMI (%)	<25.0 kg/m2	1918 (39.8)	1544 (42.3)	374 (32.0)	<0.0001
	25.0-30.0 kg/m2	2371 (49.2)	1740 (47.7)	631 (54.0)	
	>30.0 kg/m2	527 (10.9)	364 (10.0)	163 (14.0)	
Smoke Status (%)	Never smokers	3615 (74.1)	2787 (75.4)	828 (70.2)	0.0004
	Ever smokers	1261 (25.9)	909 (24.6)	352 (29.8)	
Diabetes Family history (%)	No	3408 (71.8)	2751 (76.3)	657 (57.7)	<0.0001
	Yes	1336 (28.2)	854 (23.7)	482 (42.3)	
CHD (%)	No	4179 (88.7)	3277 (91.4)	902 (79.8)	<0.0001
	Yes	535 (11.3)	307 (8.6)	228 (20.2)	
Hypertension (%)	No	3074 (67.6)	2509 (72.5)	565 (52.0)	<0.0001
	Yes	1476 (32.4)	954 (27.5)	522 (48.0)	
TC (median [IQR])		5.23 [4.64, 5.89]	5.24 [4.67, 5.89]	5.21 [4.53, 5.91]	0.0588
TG (median [IQR])		1.25 [0.91, 1.74]	1.22 [0.89, 1.68]	1.40 [0.99, 1.92]	<0.0001
HDL-C (median [IQR])		1.38 [1.15, 1.63]	1.40 [1.18, 1.66]	1.30 [1.09, 1.51]	<0.0001
LDL-C (median [IQR])		3.19 [2.69, 3.74]	3.18 [2.72, 3.73]	3.20 [2.59, 3.77]	0.3909
SBP (median [IQR])		132.00 [122.00, 143.00]	130.67 [121.00, 141.33]	136.67 [126.67, 147.67]	<0.0001
DBP (median [IQR])		74.67 [68.00, 81.00]	74.67 [68.33, 81.00]	74.33 [66.75, 81.33]	0.1171
FBG (median [IQR])		5.57 [5.22, 6.07]	5.46 [5.16, 5.81]	6.86 [6.21, 7.35]	<0.0001
OGTT (median [IQR])		7.14 [5.94, 9.16]	6.66 [5.69, 7.85]	12.23 [10.82, 13.86]	<0.0001
Lipid-lowering drug use (%)	No	4307 (89.2)	3360 (91.7)	947 (81.1)	<0.0001
	Yes	523 (10.8)	303 (8.3)	220 (18.9)	
GRS (median [IQR])		1.28 [0.91, 1.58]	1.28 [0.91, 1.58]	1.27 [0.92, 1.57]	0.3932
Genetically predicted LDL-C		3.23 [3.11, 3.35]	3.23 [3.11, 3.35]	3.23 [3.11, 3.35]	0.6578

### 3.2 Linear association between LDL-C and diabetes

We first assessed the linear association between LDL-C and diabetes in observational analysis and MR analysis. Before proceeding with the MR analysis, we evaluated whether the GRS instrumental variable predict LDL-C in our data. The F-statistic was 63.84 in the first stage of linear regression. GRS composed of 32 SNPs explained a 4.9% variation in LDL-C phenotype, the detailed information of SNPs used for constructing GRS and the association between GRS and LDL-C were presented in Supplementary Material (**Supplementary Tables 1, 2** and **Supplementary Figure 2**).

**Figure 1:**
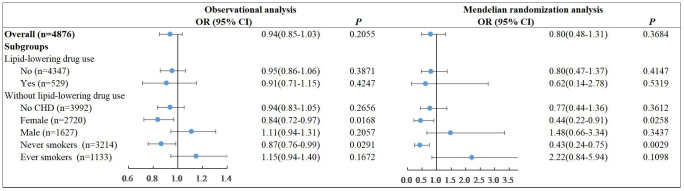
Linear associations of LDL-C with the risk of diabetes in observational analysis and MR analysis.

After adjusting for age, sex, BMI, diabetes family history, dyslipidemia, coronary heart disease (CHD), and blood pressure, we found no significant linear association between LDL-C and diabetes in all participants both in the observational analysis (*P=0.2055*) and MR analysis (*P=0.3648*) with the poor power of 0.29 (**Figure 1**). Similar results were observed in subgroups without lipid-lowering drug use. However, we found a consistently protective effect of LDL-C against diabetes among the female subjects who did not receive the lipid-lowering drug. The ORs per unit increase in LDL-C for diabetes was 0.84[95% CI, 0.72–0.97] (*P=0.0168*) in observational study. This effect was more prominent under the causal inference framework in MR analysis and suggested that increasing the overall distribution of LDL-C in females per unit would lead to an overall decrease in the risk of diabetes by 56% (95% confidence interval 9% to 78%) with the power of 0.91. We also found a similar protective effect of LDL-C against diabetes among the never smokers who did not receive the lipid-lowering drug both in observational analysis (OR=0.87, 95%CI, 0.76-0.99, *P*=0.0291) and MR analysis (OR=0.43, 95%CI, 0.24-0.75, *P*=0.0029). This was largely because the proportion of ever smokers was 64.2% among the males and 3.3% among the females (**Supplementary Table 3**). We tested the linear associations of LDL-C with the risk of diabetes with additional adjustment of smoking in MR analysis. The protective effect in females persisted (OR=0.44, 95%CI, 0.21-0.90, *P*=0.0234) (**Supplementary Table 4**).

### 3.3 Nonlinear association between LDL-C and diabetes

**Figure 2:**
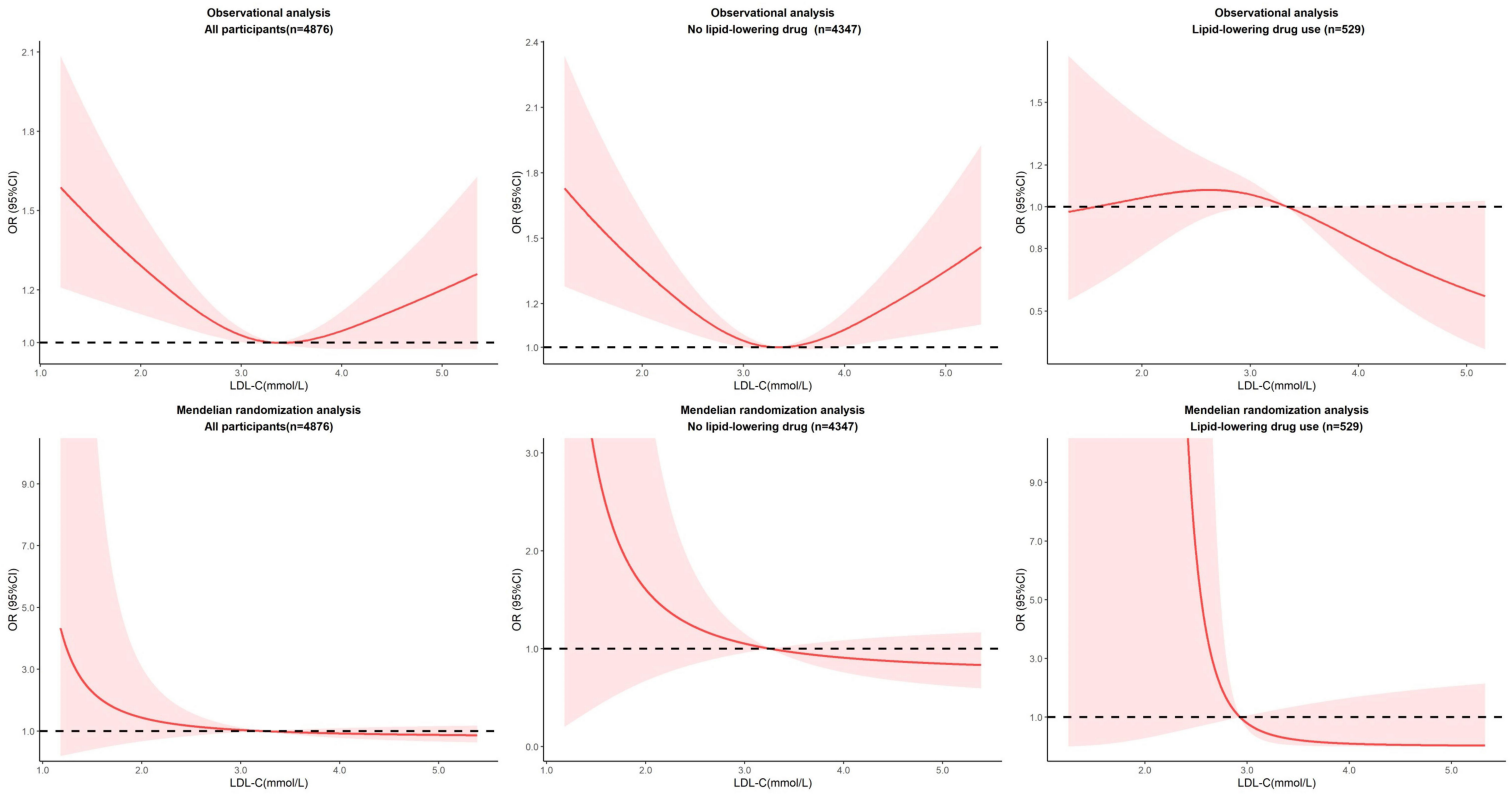
Nonlinear associations of LDL-C with the risk of diabetes in observational and MR analysis.

We subsequently assessed for nonlinear associations of LDL-C with the risk of diabetes using restricted cubic splines in observational analysis. We finally identified three nodes and non-linear relationship between LDL-C and diabetes (*P=0.0023*). The model revealed a significant U-shaped association (**Figure 2**). As the plot shows, a substantial reduction of the risk within the lower range of predicted LDL-C, which reached the lowest risk around 3.2-3.4 mmol/L and then increased thereafter (*P* for non-linearity<0.001). Below 3.2 mmol/L, the odds ratio per standard deviation higher predicted LDL-C for diabetes was 0.71 (0.58 to 0.87). Above 3.6 mmol/L, the odds ratio for genetically LDL-C for diabetes was 1.46 (1.14 to 1.86). This result persisted in subgroups without lipid-lowering drug use. Notably, among participants taking lipid-lowering drugs, there was no U-shaped association (*P* for non-linearity = 0.2144).

We performed nonlinear MR analyses by obtaining LACE estimates in centiles of instrumental variable-free exposure to further evaluate the shape of the genetic association between LDL-C and cardiovascular events. The results of all participants and subgroups of lipid-lowering drug use were summarized in **Figure 2**. We observed no evidence of a nonlinear relationship in the MR analysis (The *P* for nonlinearity in all associations between LDL-C and diabetes was >0.1). Sensitivity analyses stratified by sex show no evidence of nonlinearity (*P* for nonlinearity >0.1). And it was worth noting that although the effects were not significant, there showed a heterogeneous trend in males and females (**Supplementary Figures**).

### 3.4 Linear association between LDL-C and FBG

**Figure 3:**
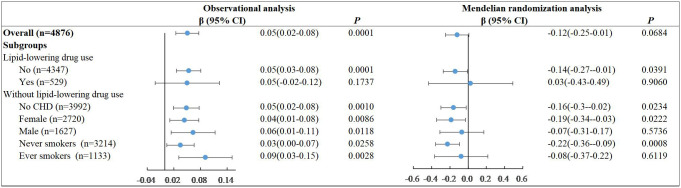
Linear associations of LDL-C and FBG in observational analysis and MR analysis.

We repeated the above analysis to explore the association between LDL-C and FBG and added the additional adjustment for hypoglycemic drug use (**Figure 3**). Under linear assumptions, the results of observational and MR studies show reverse effects. In observational analysis, per unit increase in LDL-C was associated with a 0.05 mmol/L increase in FBG (*P=0.0001*) in all participants as well as participants without lipid-lowering drug use. The results were robust across genders and CHD-free population among the non-lipid-lowering participants. In MR analysis, per unit increase in LDL-C was causally associated with a 0.14 mmol/L decrease in FBG (*P=0.0391*) among participants who weren’t taking lipid-lowering drugs. The effect was also observed in females (β=-0.19, *P=0.0222*) and in CHD-free participants (β=-0.16, *P=0.0234*). We took a sensitive analysis by excluding participants with known diabetes or hypoglycemic drug use. The effects of LDL-C on FBG were attenuated and the significance was reduced. In observational analysis, per unit increase in LDL-C was associated with a 0.03 mmol/L increase in FBG (*P=0.0161*) in all participants as well as participants without lipid-lowering drug use. In MR analysis, there were no significant association between LDL-C and FBG except among the never smokers without lipid-lowering drug use. (β=-0.06, *P*=0.0108) (**Supplementary Table 5**). The reverse effects of LDL-C against FBG in observational and MR analyses persisted though the effects were not statistically significant.

### 3.5 Nonlinear association between LDL-C and FBG

**Figure 4:**
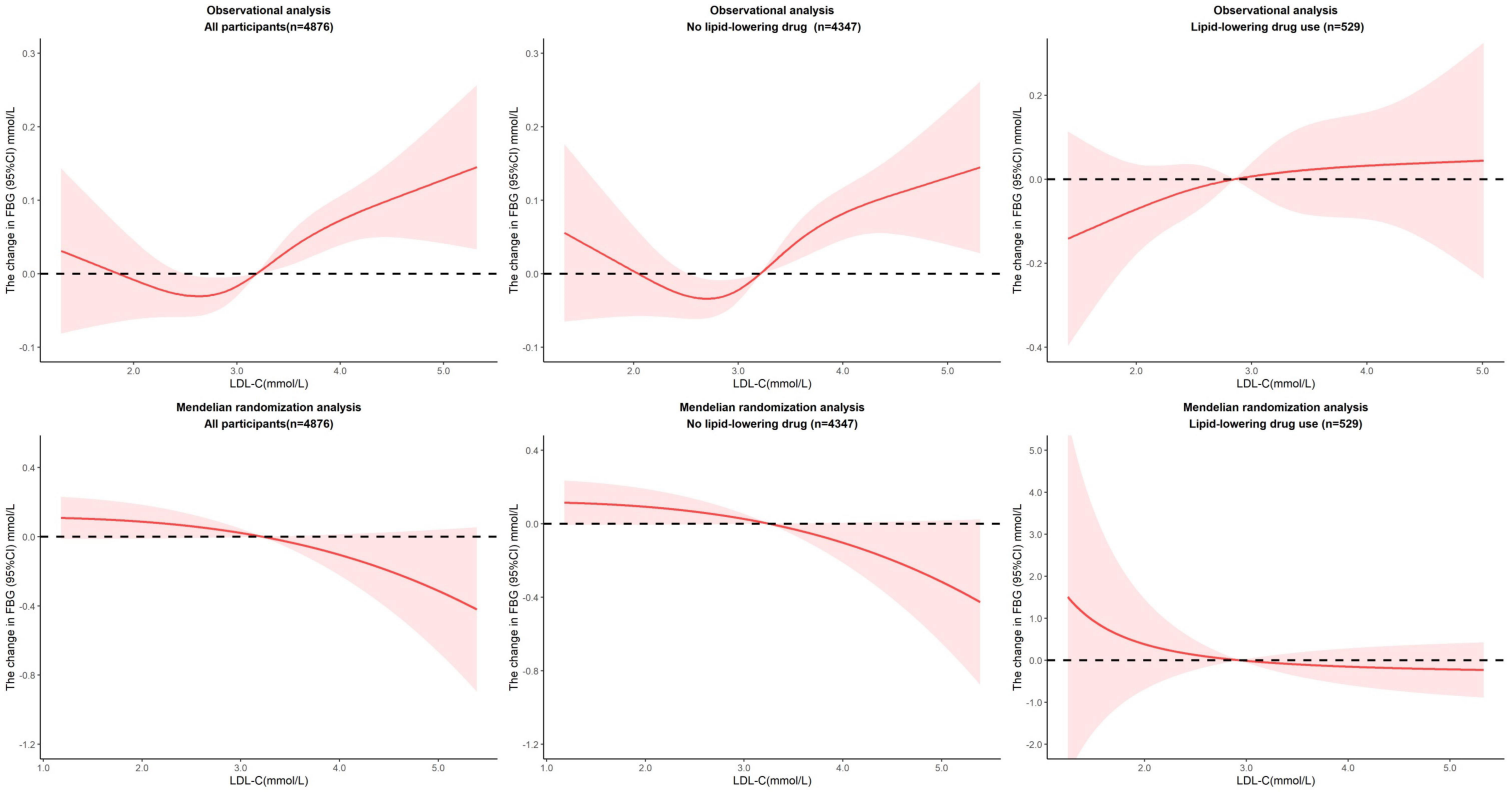
Nonlinear associations of LDL-C and FBG in observational and MR analysis.

For nonlinear association, the optimal model was fitted when we set four nodes in restricted cubic splines (*P* for non-linearity = 0.0174), which makes the relationship between LDL-C and FBG more complicated. When LDL-C fell between 2.6 mmol/L and 3.8 mmol/L, per unit increase in LDL-C was associated with a 0.29 mmol/L increase in FBG (*P=0.0069*) in all participants and 0.40 mmol/L increase in FBG (*P=0.0007*) in participants without lipid-lowering drug use. Over 3.8 mmol/L, per unit increase in LDL-C was associated with a 1.01 mmol/L decrease in FBG (*P=0.0139*) in all participants and 1.38 mmol/L decrease in FBG (*P=0.0015*) in participants without lipid-lowering drug use. We observed no evidence of a nonlinear relationship in the MR analysis (*P* value for nonlinearity in all associations between LDL-C and FBG was >0.1), but plots showed a sustained downward effect of LDL-C to FBG, though not significant (**Figure 4**). In the sensitive analysis, the effects from the observational analysis were amplified. When LDL-C fell between 2.6 mmol/L and 3.8 mmol/L, per unit increase in LDL-C was associated with a 034 mmol/L increase in FBG (*P=0.0056*) in all participants and 0.43 mmol/L increase in FBG (*P=0.0012*) in participants without lipid-lowering drug use. Over 3.8 mmol/L, per unit increase in LDL-C was associated with a 1.21 mmol/L decrease in FBG (*P=0.0090*) in all participants and 1.49 mmol/L decrease in FBG (*P=0.0019*) in participants without lipid-lowering drug use. We still did not observe any evidence of a nonlinear relationship in the MR analysis (**Supplementary Figure 5**).

## 4 Discussion

In this study, we investigated both linear and non-liner associations between LDL-C and diabetes in observational and MR studies. We found there is a complex non-linear association between LDL-C and diabetes as well as FBG in observational analysis, while the causal effect was linear in MR analysis. Under the assumption of linear association, we found a consistently protective effect of LDL-C against diabetes among the female subjects who did not receive lipid-lowering drug, while reverse effects of LDL-C against FBG in observational and MR analyses were observed, implying the potential confounding factors and disease heterogeneity.

Several guidelines on diabetes management involve LDL-C management to reduce the risk of cardiovascular disease (28–30). Our study provided suggestive evidence about the protective effect of LDL-C against diabetes, which is consistent with previous studies (8, 9, 11–13, 31). This effect is independent of the use of lipid-lowering drugs, which should be taken into consideration in the LDL-C management in diabetes. Wu et al. showed evidence of an indirect effect of lowering LDL-C on type 2 diabetes through BMI (OR 1.04 [95% CI 1.01, 1.08]) with a proportion mediated of 38% of the total effect (*P = 0.03*) (31). Richardson et al. proposed a potential explanation that the LDL particles were cleared most efficiently by the LDL pathway, and pancreas also have LDL receptors and clear LDL particles. When LDL cholesterol is increased and apoB kept constant, clearance of LDL particles by the LDL pathway is decreased, with the result that delivery of LDL particles to pancreatic islet cells could be decreased, which reduces the adverse effects on islet cell function, insulin secretion, and cell proliferation (32). The trade-off between the elevated risk of subsequent diabetes from the combination of lipid-lowering drug use and LDL-C reduction and the reduced cardiovascular risk needs to be carefully balanced, especially there appear to be important sexual dimorphisms in its relationships with blood glucose management, with greater impact in women. Sexual dimorphisms were ever reported in lipid treatment response. Hamrefors et al. showed a genetic score of LDL and HDL-associated single nucleotide polymorphisms were directly correlated with more pronounced fluvastatin-induced HDL increase, explaining 5.9-11.6% of the variance in treatment response in women and no such associations in men. Previous evidence has also suggested sex-specific heritability of lipid traits (33). Teslovich et al. re-analyzed the GWAS for the four lipid traits separately in women (n = 63,274) and in men (n = 38,514) and found four loci with significant heterogeneity of effect size (P< 0.0005) between the gender (34). Two loci (*KLF14* and *ABCA8*) showed female-specific association with TG and LDL-C, respectively (34). The underlying mechanisms may involve estrogen metabolism and methylation processes. Huang et al. reported PvuII restriction enzyme polymorphisms of estrogen receptor alpha (*ESR α*) gene (also named *ESR1*) increased susceptibility to type 2 diabetes mellitus and may also impact serum lipid metabolism in Chinese Guangzhou women (35). A study including 1143 rural residents recruited randomly from Henan Province China explored the potential association between the ESRα promoter methylation, lipid metabolism, and the risk of type 2 diabetes mellitus and found that the ESRα promoter methylation levels were negatively associated with HDL-C levels whether gender stratification was performed (*P< 0.05*) and positively correlated with LDL-C in men (*P< 0.05*) (36, 37). The existing vitro experiments suggested that the variant allele (C at IVS1–401) of *ESR1* might provide a functionally significant binding site for myb transcription factor and Herrington et al. assumed that the estrogen receptor-mediated pathway may play a role in HDL cholesterol response to statin treatment, as reported in hormone replacement therapy (38, 39). However, it is not clear whether this mechanism also plays a role in LDL cholesterol response. Inhibition of 3-hydroxy-3-methylglutaryl-CoA reductase (HMGCR), which is the intended drug target to reduce LDL cholesterol concentration, was significantly associated with lower odds of epithelial ovarian cancer (40). However, this research did not indicate further underlying mechanisms of LDL-C metabolism interaction with estrogen. Brüning et al. confirmed that estradiol- and tamoxifen-stimulated expression depends on an intact repeat 3 in the LDL receptor promoter and estradiol- and tamoxifen-stimulated binding of nuclear proteins to repeat 3 (bp -56 to bp -36) of the LDL receptor promoter by transient transfection experiments (37).

It’s worth noting that we observed a complex non-linear association between LDL-C and diabetes as well as FBG in observational analysis, but no evidence of a causal nonlinear association in the MR analysis. This means more cautious interpretation needs to be considered in observational studies, especially when the prevalent non-communicable diseases globally have an evident close co-morbid trend. Shared risk factors may induce more confounding factors and heterogeneity may induce information bias that masks true associations. It is a good attempt to supplement more accurate biological phenotypes as secondary outcomes in observational studies to reveal the intricate mechanisms. It appeals for an integrated and prospective interrogation into their intercorrelation. In this study, we observed the reverse effects of LDL-C against FBG in observational and MR analyses. This may be attributed to the heterogeneity of diabetes. Klimentidis et al. found that levels of circulating LDL-C were negatively associated with type 2 diabetes prevalence (odds ratio 0.41 [95% CI 0.39, 0.43] per mmol/L unit of LDL-C), despite positive associations of circulating LDL-C with HbA1c and BMI (13). They speculated it as a threshold effect, whereby the etiology of “normal” HbA1c variation is somewhat distinct from the etiology of crossing into overt type 2 diabetes. From our overall results, we believed that residual confounding due to the undetectable confounding factors was more likely to be the culprit. It is also inevitable to assess the effect of LDL-C on the function of insulin secretion and insulin resistance. Therefore, the relationship between the specific diabetes-related phenotypes and LDL-C needs to be carefully evaluated in future observational studies and causal inference.

Our study is subject to several limitations. First, we used prevalent diabetes instead of incident diabetes in the FISSIC study. Although MR analyses were used to circumvent potential confounding and reverse causation, the results from observational studies are not as direct as that from prospective studies, which limits inferences and directions related to the causality. As incident diabetes cases develop in further research, it will be important to examine the association of LDL-C at baseline with incident diabetes. Second, we did not cover other specific diabetes-related phenotypes, such as HbA1c, a long-term glycaemic control indicator that helps distinguish patients with poor long-term glycaemic control from those with stress hyperglycemia, or HOMA-IR, the most popular indicator to qualify the degree of insulin resistance. In addition, although the participants in our study were aged ≥40 years, which avoided the confounding of type 1 diabetes to some extent, we could not further refine diabetes into more precise subtypes, but only generalized the cases to type 2 diabetes. And there were not sufficient data for the other complications. This limited our further exploration of underlying mechanisms by which LDL-C impacts the risk of diabetes, especially when diabetes is a heterogeneous disease overtly. Finally, our MR analysis was underpowered for the association of LDL with FBG and diabetes but was adequately powered for the association of LDL with diabetes among female subjects who did not receive the lipid-lowering drug.

In conclusion, we verified the liner protective effect of LDL-C against diabetes among the female subjects who did not receive lipid-lowering drugs. Non-linear associations between LDL-C against diabetes in observational analysis are not causal, and prospective cohort study is welcomed to address this issue and to further explore the underlying mechanisms.

## Data availability statement

We estimated statistic power for the MR studies using the online website (https://shiny.cnsgenomics.com/mRnd/). Nonlinear MR available in the nlmr R package (https://github.com/jrs95/nlmr).

## Ethics statement

The studies involving human participants were reviewed and approved by the Ethics Committee of the Peking University Health Science Center (Approval number: IRB00001052-13027). The patients/participants provided their written informed consent to participate in this study.

## Author contributions

DC conceived the study, undertook project leadership. In addition, DC, TW, and YW were guarantors of this work. YM wrote the first draft of the manuscript, analyzed data and interpreted the results. ZZ, XL, KD, and HX were involved in the data collection. All authors contributed to the drafting and critical revision of the manuscript. All authors contributed to the article and approved the submitted version.

## Funding

This work was supported by the National Natural Science Foundation of China (No.81872692; NO.82073642).

## Acknowledgments

We thank the research participants and researchers for their help in data collection and methodology.

## Conflict of interest

The authors declare that the research was conducted in the absence of any commercial or financial relationships that could be construed as a potential conflict of interest.

## Publisher’s note

All claims expressed in this article are solely those of the authors and do not necessarily represent those of their affiliated organizations, or those of the publisher, the editors and the reviewers. Any product that may be evaluated in this article, or claim that may be made by its manufacturer, is not guaranteed or endorsed by the publisher.

## Abbreviations

FBG, Fasting blood glucose; OGTT, Oral glucose tolerance test; LDL-C, Low-density lipoprotein cholesterol; MetS, Metabolic syndrome; HDL-C, High-density lipoprotein cholesterol; MR, Mendelian randomization; LACE, Localized average causal effect; HOMA-IR, Homeostatic model assessment for insulin resistance; CHD, Coronary heart disease; BMI, Body mass index; IQR, Interquartile range; TC, Total cholesterol; TG, Total triglycerides; SBP, Systolic blood pressure; DBP, Diastolic blood pressure; LD, linkage disequilibrium; SNP, Single nucleotide polymorphisms; GWAS, Genome-wide association study; GRS, Genetic risk score; NCP, non-centrality parameter.
