# Supplementary material for: Linear and nonlinear analyses of the association between low–density lipoprotein cholesterol and diabetes: The spurious U–curve in observational study
Source: Front Endocrinol (Lausanne). 2022 Nov 17;13:1009095. doi: 10.3389/fendo.2022.1009095 (PMC9714469; doi:10.3389/fendo.2022.1009095)
Supplement: Supplementary file 1 [file DataSheet_1.pdf]

Supplementary Material

1 Supple Supplementary data

Power calculation

In Brion et al research, the statistical power in Mendelian randomization studies could be calculated as: $Power = 1 - P(\chi^{2'}_{df,NCP} > \chi^{2}_{df,1-\alpha})$

where $\chi^{2'}_{df,NCP}$ is a random variable from a non-central χ2 distribution with df degrees of freedom and $\chi^{2}_{df,1-\alpha}$ is the threshold of a central χ2 distribution for a type-I error rate of α. NCP (non-centrality parameter) is a function of the asymptotic mean and variance of the instrumental variables (IVs) estimator, which is proposed by Brion et al to test whether the two-stage least squares (2SLS) IV regression coefficient is zero.

For continuous outcomes,

$$NCP = [n\,\rho^{2}_{xz}\,\sigma^{2}_{x}]\,\{\beta_{yx} + (\beta_{OLS} - \beta_{yx})\sigma^{2}_{x}\ /\ (n\,\rho^{2}_{xz})\}^{2}\ /\ \{\sigma^{2}_{y} - \sigma^{2}_{x}\beta_{yx}[2\beta_{OLS} - \beta_{yx}]\}$$

with β_{yx} the causal effect of X on Y (i.e. the parameter of interest) and β_{OLS} the asymptotic value of the ordinary least squares (OLS) estimator of the effect of X and Y. In the presence of XY confounding, these two population parameters are not the same; n is the experimental sample size, ρ^{2}_{xz} the population value for the proportion of variance in the exposure variable explained by the genetic predictor, and σ^{2}_{y} and σ^{2}_{x} the variances of Y and X, respectively.

For binary outcomes,

$$NCP = \frac{b_{MR}^2}{b_{MR}} = NR_{xz}^2 \frac{K^2(\frac{OR}{1+K(OR-1)} - 1)^2}{K(1-K) - b_{01}^2}$$

where K the proportion of cases in the (intended) study, N the total sample size, OR the true odds ratio of the outcome variable per standard deviation of the exposure variable, R_{xz}^2 the proportion of variance in exposure variable explained by SNPs. With input variables OR and K, the regression coefficient is derived on the observed scale:

$$b_{01} = K(\frac{OR}{1+K(OR-1)} - 1)$$

2 Supplementary Tables

Supplementary table 1 Information of SNPs used for constructing GRS

RSID	CHR	POS	N	EA	NEA	EAF	BETA	SE	P	TYPE	GENE
rs611917	1	109815252	22233	A	G	0.9326	0.2045	0.0201	8.06E-24	intronic	CELSR2
rs12740374	1	109817590	20485	T	G	0.0635	-0.2025	0.0206	8.52E-23	UTR3	CELSR2
rs629301	1	109818306	21444	T	G	0.9332	0.1986	0.0196	2.16E-24	UTR3	CELSR2
rs646776	1	109818530	20479	T	C	0.9357	0.1982	0.0204	1.31E-22	downstream	CELSR2
rs602633	1	109821511	22228	T	G	0.0651	-0.1879	0.0201	2.35E-20	downstream	PSRC1
rs599839	1	109822166	31727	A	G	0.9307	0.1877	0.0162	9.84E-31	downstream	PSRC1
rs599839	1	109822166	31727	A	G	0.9307	0.1877	0.0162	9.84E-31	downstream	PSRC1
rs505151	1	55529187	29619	A	G	0.9442	-0.104	0.0184	2.40E-08	nonsynonymous	PCSK9
rs1559401	16	72011181	31721	T	G	0.7184	0.0685	0.0091	8.87E-14	unknown	unknown
rs7185272	16	72013797	31725	C	G	0.7187	0.0692	0.0091	4.81E-14	unknown	unknown
rs2738446	19	11227326	29672	C	G	0.8362	-0.0683	0.0114	3.26E-09	intronic	LDLR
rs519113	19	45376284	23752	C	G	0.8372	-0.0893	0.0128	3.51E-12	intronic	PVRL2
rs2075650	19	45395619	17155	A	G	0.9029	-0.1676	0.0186	7.16E-20	intronic	TOMM40
rs405509	19	45408836	17155	T	G	0.6752	0.1624	0.012	4.08E-42	upstream	APOE
rs7412	19	45412079	10133	T	C	0.0847	-0.5934	0.0266	8.20E-107	nonsynonymous	APOE
rs439401	19	45414451	17154	T	C	0.5666	0.0725	0.0113	1.09E-10	intergenic	APOE(dist=1801), APOC1(dist=3470)

rs445925	19	45415640	15874	A	G	0.0937	-0.5219	0.0214	1.79E-129	intergenic	APOE(dist=2990), APOC1(dist=2281)
rs11902417	2	21198900	31732	A	G	0.7228	0.0547	0.0091	2.02E-09	intergenic	C2orf43(dist=176073), APOB(dist=25401)
rs6544366	2	21204025	31731	T	G	0.723	0.0563	0.0092	6.89E-10	intergenic	C2orf43(dist=181198), APOB(dist=20276)
rs6754295	2	21206183	23191	T	G	0.2905	-0.061	0.0106	9.93E-09	intergenic	C2orf43(dist=183356), APOB(dist=18118)
rs1042034	2	21225281	22157	T	C	0.274	-0.0639	0.0113	2.13E-08	nonsynonymous	APOB
rs676210	2	21231524	23190	A	G	0.7296	0.0613	0.0109	2.44E-08	nonsynonymous	APOB
rs7703051	5	74625487	31731	A	C	0.5273	0.0768	0.0082	5.28E-21	intergenic	ANKRD31(dist=92784), HMGCR(dist=7506)
rs12654264	5	74648603	31724	A	T	0.4745	-0.0784	0.0082	7.16E-22	intronic	HMGCR
rs3846662	5	74651084	31721	A	G	0.473	-0.0778	0.0082	1.65E-21	intronic	HMGCR
rs3846663	5	74655726	31731	T	C	0.5248	0.0789	0.0082	4.30E-22	intronic	HMGCR
rs5744680	5	74879890	31637	A	G	0.5277	0.0769	0.0084	4.06E-20	intronic	POLK
rs40060	5	74967386	31659	T	C	0.367	-0.0626	0.0085	1.50E-13	intergenic	POLK(dist=71740), POC5(dist=2638)
rs6982636	8	126479315	31415	A	G	0.5634	-0.0467	0.0083	1.20E-08	intergenic	TRIB1(dist=28671), LOC100130231(dist=455452)
rs2954029	8	126490972	31698	A	T	0.4408	0.0479	0.0083	4.27E-09	intergenic	TRIB1(dist=40328), LOC100130231(dist=443795)
rs651007	9	136153875	31726	T	C	0.3464	0.0571	0.0097	1.93E-09	intergenic	ABO(dist=3245), SURF6(dist=43677)
rs579459	9	136154168	31677	T	C	0.6493	-0.0568	0.0096	1.73E-09	intergenic	ABO(dist=3538), SURF6(dist=43384)

Supplementary table 2 Association of GRS on LDL-C

	Estimate	SE	t value	P
(Intercept)	-0.18	0.43	-0.42	6.72E-01
GRS	0.22	0.02	11.34	2.00E-16
Sex	0.20	0.02	8.61	2.00E-16
AGE	0.10	0.01	6.84	9.10E-12
Age*Age	0.00	0.00	-6.55	6.57E-11

Supplementary table 3 Distribution of the smoking and sex among the participants without lipid-lowering drug use

	ALL	Never smoker	Ever smoker
Male	1627	583	1044
Female	2720	2631	89

Supplementary table 4 Linear associations of LDL-C with the risk of diabetes with additional adjustment of smoking in MR analyses

	**OR**	**95%CI_low**	**95%CI_up**	***P***
**Overall (n=4876)**	0.80	0.48	1.31	0.3696
**Subgroups**				
Lipid-lowering drug use				
No (n=4347)	0.80	0.47	1.37	0.4128
Yes (n=529)	0.60	0.13	2.72	0.5117
Without lipid-lowering drug use				
No CHD (n=3992)	0.77	0.44	1.36	0.3610
Female (n=2720)	0.44	0.21	0.90	0.0234
Male (n=1627)	1.47	0.66	3.33	0.3475

Supplementary table 5 Linear associations of LDL-C and FBG among participants without diabetes nor hypoglycemic drug use in observational analyses and MR analyses

	**Observational analyses**				**Mendelian randomization analyses**			
	β	95%CI_Low	95%CI_Up	P	β	95%CI_Low	95%CI_Up	P
**Overall (n=3696)**	0.03	0.00	0.05	0.0161	-0.09	-0.20	0.02	0.0997
**Subgroups**								
Lipid-lowering drug use								
No (n=3390)	0.03	0.01	0.05	0.0129	-0.08	-0.19	0.03	0.1512
Yes (n=306)	0.00	-0.06	0.06	0.8969	-0.09	-0.53	0.35	0.6758
Without lipid-lowering drug use								
No CHD (n=3171)	0.03	0.01	0.06	0.0132	-0.07	-0.19	0.04	0.2090
Female (n=2204)	0.04	0.01	0.06	0.0121	-0.12	-0.26	0.01	0.0678

Male (n=1186)	0.01	-0.03	0.05	0.6703	-0.01	-0.21	0.19	0.9022
Never smokers (n=2554)	0.03	0.00	0.06	0.0207	-0.15	-0.26	-0.03	0.0108
Ever smokers (n=836)	0.01	-0.04	0.06	0.7727	-0.06	-0.29	0.16	**2.5877**

Supplementary table 6 Additional summary demographic information of participants in observational analysis (stratified by quartiles of LDL-C (mmol/L))

		**The quartiles of LDL-C (mmol/L)**				
		**[1.18,2.69]**	**(2.69,3.19]**	**(3.19,3.74]**	**(3.74,5.39]**	**p**
No of participants		1228	1236	1201	1211	
Sex (%)	Male	549 (44.7)	458 (37.1)	438 (36.5)	369 (30.5)	<0.0001
	Female	679 (55.3)	778 (62.9)	763 (63.5)	842 (69.5)	
Age at baseline (median [IQR], years)		56.00 [49.00, 63.00]	55.00 [51.00, 61.00]	56.00 [51.00, 62.00]	56.00 [52.00, 62.00]	0.0078
BMI (median [IQR], kg/m2)		25.58 [23.17, 27.89]	25.64 [23.62, 27.72]	26.00 [24.12, 28.21]	26.13 [24.14, 28.38]	<0.0001
BMI (%)	<25.0 kg/m2	541 (44.1)	527 (42.6)	448 (37.3)	422 (34.8)	<0.0001
	25.0-30.0 kg/m2	556 (45.3)	586 (47.4)	628 (52.3)	633 (52.3)	
	>30.0 kg/m2	131 (10.7)	123 (10.0)	125 (10.4)	156 (12.9)	
Smoke Status (%)	Never smokers	871 (70.9)	919 (74.4)	884 (73.6)	941 (77.7)	0.0020
	Ever smokers	357 (29.1)	317 (25.6)	317 (26.4)	270 (22.3)	
Diabetes Family history (%)	No	862 (70.2)	921 (74.5)	860 (71.6)	865 (71.4)	0.1053
	Yes	366 (29.8)	315 (25.5)	341 (28.4)	346 (28.6)	
CHD (%)	No	1033 (84.1)	1116 (90.3)	1080 (89.9)	1088 (89.8)	<0.0001
	Yes	195 (15.9)	120 (9.7)	121 (10.1)	123 (10.2)	
Hypertension (%)	No	873 (71.1)	936 (75.7)	799 (66.5)	685 (56.6)	<0.0001
	Yes	355 (28.9)	300 (24.3)	402 (33.5)	526 (43.4)	
TC (median [IQR])		4.21 [3.85, 4.56]	4.93 [4.70, 5.19]	5.48 [5.24, 5.73]	6.35 [6.01, 6.77]	<0.0001
TG (median [IQR])		1.03 [0.74, 1.55]	1.14 [0.84, 1.66]	1.35 [1.03, 1.84]	1.57 [1.21, 2.08]	<0.0001
HDL-C (median [IQR])		1.38 [1.09, 1.68]	1.39 [1.15, 1.67]	1.37 [1.16, 1.62]	1.39 [1.22, 1.61]	0.0585
LDL-C (median [IQR])		2.32 [2.03, 2.53]	2.95 [2.84, 3.07]	3.45 [3.32, 3.58]	4.14 [3.92, 4.49]	<0.0001
SBP (median [IQR])		130.83 [121.00, 142.33]	131.33 [121.00, 142.33]	133.33 [123.00, 143.33]	133.67 [123.83, 144.50]	<0.0001
DBP (median [IQR])		73.67 [67.00, 80.00]	74.33 [67.67, 80.67]	75.67 [68.33, 82.00]	75.00 [69.00, 82.00]	<0.0001

FBG (median [IQR])		5.60 [5.19, 6.26]	5.55 [5.19, 6.09]	5.65 [5.26, 6.24]	5.68 [5.32, 6.29]	<0.0001
OGTT (median [IQR])		7.30 [5.91, 9.77]	6.99 [5.89, 9.02]	7.25 [6.02, 9.57]	7.57 [6.20, 10.02]	<0.0001
Lipid-lowering drug use (%)	No	996 (81.1)	1136 (91.9)	1096 (91.3)	1119 (92.4)	<0.0001
	Yes	232 (18.9)	100 (8.1)	105 (8.7)	92 (7.6)	
Diabetes (%)	No	892 (72.6)	985 (79.7)	914 (76.1)	905 (74.7)	0.0005
	Yes	336 (27.4)	251 (20.3)	287 (23.9)	306 (25.3)	

Supplementary table 7 Additional summary demographic information of participants without lipid-lowering drug use in observational analysis (stratified by quartiles of LDL-C (mmol/L))

		**The quartile of LDL-C (mmol/L) (without lipid-lowering drug use)**				
		**[1.18,2.69]**	**(2.69,3.19]**	**(3.19,3.74]**	**(3.74,5.39]**	**p**
No of participants		996	1136	1096	1119	
Sex (%)	Male	437 (43.9)	433 (38.1)	411 (37.5)	346 (30.9)	<0.0001
	Female	559 (56.1)	703 (61.9)	685 (62.5)	773 (69.1)	
Age at baseline (median [IQR], years)		54.00 [49.00, 62.00]	55.00 [50.00, 61.00]	55.00 [50.00, 61.25]	56.00 [52.00, 62.00]	<0.0001
BMI (median [IQR], kg/m2)		25.39 [22.94, 27.64]	25.53 [23.59, 27.68]	25.95 [24.09, 28.09]	26.04 [24.11, 28.31]	<0.0001
BMI (%)	<25.0 kg/m2	460 (46.2)	502 (44.2)	418 (38.1)	394 (35.2)	<0.0001
	25.0-30.0 kg/m2	432 (43.4)	524 (46.1)	570 (52.0)	583 (52.1)	
	>30.0 kg/m2	104 (10.4)	110 (9.7)	108 (9.9)	142 (12.7)	
Smoke Status (%)	Never smokers	709 (71.2)	837 (73.7)	803 (73.3)	865 (77.3)	0.0130
	Ever smokers	287 (28.8)	299 (26.3)	293 (26.7)	254 (22.7)	
Diabetes Family history (%)	No	718 (72.1)	852 (75.0)	790 (72.1)	797 (71.2)	0.2005
	Yes	278 (27.9)	284 (25.0)	306 (27.9)	322 (28.8)	
CHD (%)	No	907 (91.1)	1061 (93.4)	1006 (91.8)	1018 (91.0)	0.1329
	Yes	89 (8.9)	75 (6.6)	90 (8.2)	101 (9.0)	
Hypertension (%)	No	845 (84.8)	932 (82.0)	795 (72.5)	681 (60.9)	<0.0001
	Yes	151 (15.2)	204 (18.0)	301 (27.5)	438 (39.1)	
TC (median [IQR])		4.24 [3.88, 4.58]	4.93 [4.70, 5.18]	5.47 [5.24, 5.72]	6.33 [6.00, 6.76]	<0.0001
TG (median [IQR])		0.98 [0.71, 1.38]	1.12 [0.82, 1.61]	1.33 [1.01, 1.81]	1.57 [1.19, 2.07]	<0.0001
HDL-C (median [IQR])		1.41 [1.11, 1.72]	1.40 [1.16, 1.68]	1.37 [1.16, 1.62]	1.39 [1.22, 1.61]	0.1832

LDL-C (median [IQR])		2.34 [2.07, 2.53]	2.95 [2.84, 3.07]	3.45 [3.31, 3.58]	4.13 [3.92, 4.48]	<0.0001
SBP (median [IQR])		130.33 [120.33, 141.00]	131.33 [121.00, 143.00]	133.33 [123.00, 143.00]	133.33 [123.67, 144.83]	<0.0001
DBP (median [IQR])		74.00 [67.00, 80.33]	74.67 [68.00, 80.67]	75.67 [68.67, 82.00]	75.00 [69.33, 82.00]	<0.0001
FBG (median [IQR])		5.54 [5.15, 6.12]	5.52 [5.18, 6.01]	5.63 [5.26, 6.19]	5.69 [5.33, 6.28]	<0.0001
OGTT (median [IQR])		7.00 [5.73, 9.11]	6.92 [5.81, 8.77]	7.14 [5.96, 9.44]	7.54 [6.19, 10.02]	<0.0001
Diabetes (%)	No	764 (76.7)	934 (82.2)	852 (77.7)	840 (75.1)	0.0004
	Yes	232 (23.3)	202 (17.8)	244 (22.3)	279 (24.9)	

Supplementary table 8 Additional summary demographic information of participants in observational analysis (stratified by quartiles of GRS)

		**The quartile of GRS (mmol/L)**				
		**[-1.68,0.906]**	**(0.906,1.28]**	**(1.28,1.58]**	**(1.58,2.31]**	**p**
No of participants		1219	1219	1220	1218	
Sex (%)	Male	431 (35.4)	442 (36.3)	487 (39.9)	454 (37.3)	0.1071
	Female	788 (64.6)	777 (63.7)	733 (60.1)	764 (62.7)	
Age at baseline (median [IQR], years)		55.00 [50.00, 62.00]	56.00 [51.00, 62.00]	56.00 [51.00, 62.00]	56.00 [51.00, 62.00]	0.6749
BMI (median [IQR], kg/m2)		25.71 [23.62, 27.80]	25.81 [23.82, 28.21]	25.98 [24.01, 28.19]	25.81 [23.79, 28.07]	0.1940
BMI (%)	<25.0 kg/m2	508 (41.7)	469 (38.5)	465 (38.1)	496 (40.7)	0.4008
	25.0-30.0 kg/m2	593 (48.6)	611 (50.1)	612 (50.2)	587 (48.2)	
	>30.0 kg/m2	118 (9.7)	139 (11.4)	143 (11.7)	135 (11.1)	
Smoke Status (%)	Never smokers	928 (76.1)	906 (74.3)	874 (71.6)	907 (74.5)	0.0865
	Ever smokers	291 (23.9)	313 (25.7)	346 (28.4)	311 (25.5)	
Diabetes Family history (%)	No	856 (70.2)	873 (71.6)	900 (73.8)	879 (72.2)	0.2721
	Yes	363 (29.8)	346 (28.4)	320 (26.2)	339 (27.8)	
CHD (%)	No	1088 (89.3)	1091 (89.5)	1071 (87.8)	1067 (87.6)	0.3269
	Yes	131 (10.7)	128 (10.5)	149 (12.2)	151 (12.4)	
Hypertension (%)	No	865 (71.0)	848 (69.6)	819 (67.1)	761 (62.5)	<0.0001
	Yes	354 (29.0)	371 (30.4)	401 (32.9)	457 (37.5)	
TC (median [IQR])		5.06 [4.51, 5.72]	5.21 [4.62, 5.84]	5.30 [4.70, 6.00]	5.34 [4.78, 6.01]	<0.0001
TG (median [IQR])		1.29 [0.91, 1.80]	1.28 [0.92, 1.84]	1.29 [0.89, 1.81]	1.28 [0.93, 1.81]	0.9591

HDL-C (median [IQR])		1.42 [1.18, 1.68]	1.38 [1.14, 1.64]	1.38 [1.14, 1.63]	1.36 [1.16, 1.62]	0.0063
LDL-C (median [IQR])		3.03 [2.51, 3.54]	3.15 [2.68, 3.68]	3.28 [2.73, 3.85]	3.30 [2.84, 3.86]	<0.0001
SBP (median [IQR])		132.00 [122.33, 143.67]	132.33 [122.00, 143.00]	132.67 [123.00, 143.00]	132.00 [121.67, 143.00]	0.5312
DBP (median [IQR])		75.33 [68.00, 81.67]	74.00 [67.00, 81.00]	75.00 [68.67, 81.33]	74.33 [68.33, 81.00]	0.1220
FBG (median [IQR])		5.64 [5.25, 6.24]	5.64 [5.25, 6.23]	5.62 [5.22, 6.25]	5.59 [5.24, 6.13]	0.4529
OGTT (median [IQR])		7.22 [6.03, 9.48]	7.47 [5.98, 9.82]	7.14 [5.97, 9.63]	7.23 [6.00, 9.38]	0.5172
Lipid-lowering drug use (%)	No	1111 (91.1)	1084 (88.9)	1097 (89.9)	1055 (86.6)	0.0031
	Yes	108 (8.9)	135 (11.1)	123 (10.1)	163 (13.4)	
Diabetes (%)	No	931 (76.4)	908 (74.5)	910 (74.6)	947 (77.8)	0.1821
	Yes	288 (23.6)	311 (25.5)	310 (25.4)	271 (22.2)	

Supplementary table 9 Additional summary demographic information of participants without lipid-lowering drug use in observational analysis (stratified by quartiles of GRS)

		**The quartile of GRS (without lipid-lowering drug use)**				
		**[-1.68,0.906]**	**(0.906,1.28]**	**(1.28,1.58]**	**(1.58,2.31]**	**p**
No of participants		1111	1084	1097	1055	
Sex (%)	Male	392 (35.3)	399 (36.8)	433 (39.5)	403 (38.2)	0.2050
	Female	719 (64.7)	685 (63.2)	664 (60.5)	652 (61.8)	
Age at baseline (median [IQR], years)		55.00 [50.00, 62.00]	55.00 [50.00, 61.00]	55.00 [51.00, 61.00]	56.00 [50.00, 62.00]	0.8163
BMI (median [IQR], kg/m2)		25.60 [23.59, 27.74]	25.76 [23.68, 28.11]	25.88 [23.83, 28.04]	25.64 [23.63, 27.78]	0.2411
BMI (%)	<25.0 kg/m2	473 (42.6)	422 (38.9)	430 (39.2)	449 (42.6)	0.2753
	25.0-30.0 kg/m2	531 (47.8)	538 (49.6)	538 (49.0)	502 (47.6)	
	>30.0 kg/m2	107 (9.6)	124 (11.4)	129 (11.8)	104 (9.9)	
Smoke Status (%)	Never smokers	854 (76.9)	798 (73.6)	783 (71.4)	779 (73.8)	0.0328
	Ever smokers	257 (23.1)	286 (26.4)	314 (28.6)	276 (26.2)	
Diabetes Family history (%)	No	785 (70.7)	793 (73.2)	815 (74.3)	764 (72.4)	0.2751
	Yes	326 (29.3)	291 (26.8)	282 (25.7)	291 (27.6)	
CHD (%)	No	1019 (91.7)	1001 (92.3)	1004 (91.5)	968 (91.8)	0.9088
	Yes	92 (8.3)	83 (7.7)	93 (8.5)	87 (8.2)	
Hypertension (%)	No	857 (77.1)	836 (77.1)	810 (73.8)	750 (71.1)	0.0022

	Yes	254 (22.9)	248 (22.9)	287 (26.2)	305 (28.9)	
TC (median [IQR])		5.07 [4.53, 5.72]	5.23 [4.67, 5.86]	5.34 [4.77, 6.02]	5.39 [4.83, 6.03]	<0.0001
TG (median [IQR])		1.26 [0.90, 1.77]	1.26 [0.91, 1.80]	1.25 [0.88, 1.80]	1.25 [0.92, 1.77]	0.9755
HDL-C (median [IQR])		1.42 [1.19, 1.68]	1.39 [1.15, 1.65]	1.39 [1.15, 1.64]	1.37 [1.17, 1.63]	0.0236
LDL-C (median [IQR])		3.04 [2.54, 3.55]	3.18 [2.74, 3.70]	3.30 [2.81, 3.88]	3.36 [2.89, 3.88]	<0.0001
SBP (median [IQR])		132.33 [122.33, 143.83]	132.33 [121.67, 143.33]	132.33 [123.00, 142.67]	131.33 [121.33, 143.00]	0.6192
DBP (median [IQR])		75.67 [68.00, 82.00]	74.33 [67.33, 81.00]	75.00 [68.67, 81.33]	74.33 [68.33, 81.00]	0.2383
FBG (median [IQR])		5.61 [5.23, 6.19]	5.62 [5.23, 6.18]	5.60 [5.20, 6.21]	5.57 [5.22, 6.06]	0.3735
OGTT (median [IQR])		7.11 [5.95, 9.26]	7.34 [5.91, 9.62]	7.04 [5.92, 9.39]	7.07 [5.91, 9.16]	0.4563
Diabetes (%)	No	867 (78.0)	834 (76.9)	840 (76.6)	849 (80.5)	0.1230
	Yes	244 (22.0)	250 (23.1)	257 (23.4)	206 (19.5)	

3 Supplementary Figures

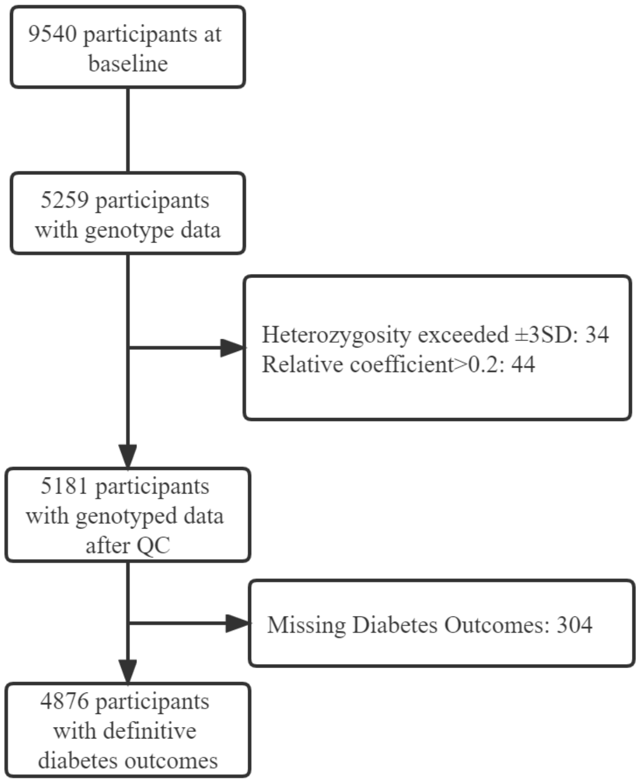

Supplementary Figure 1. Distribution of GRS on LDL-C Quintile.

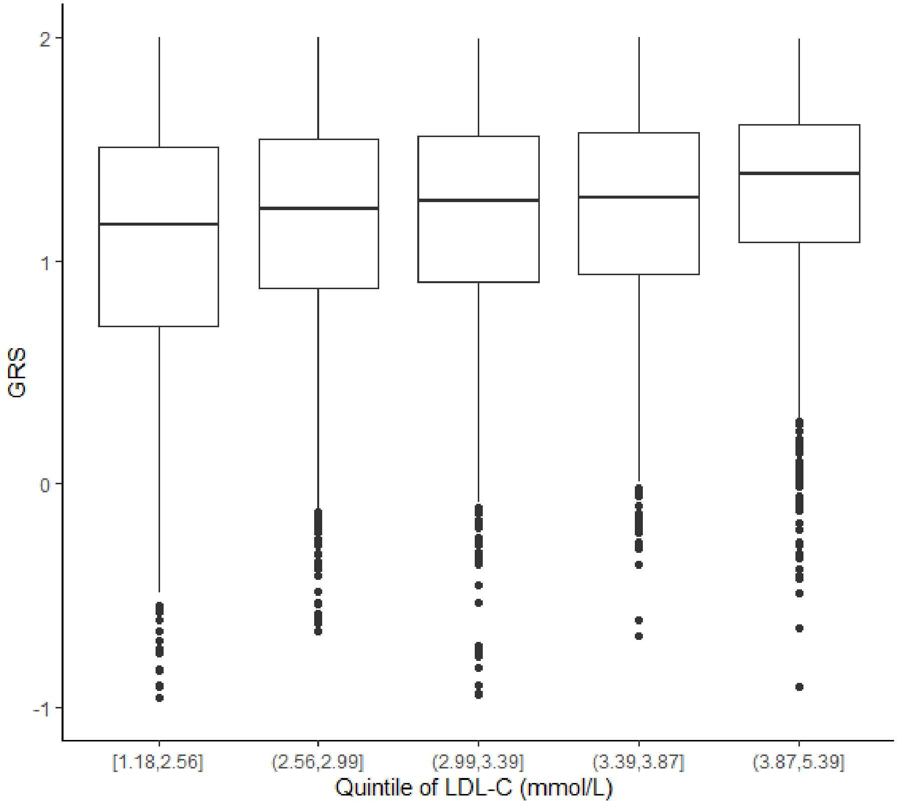

Supplementary Figure 2. Distribution of GRS on LDL-C Quintile.

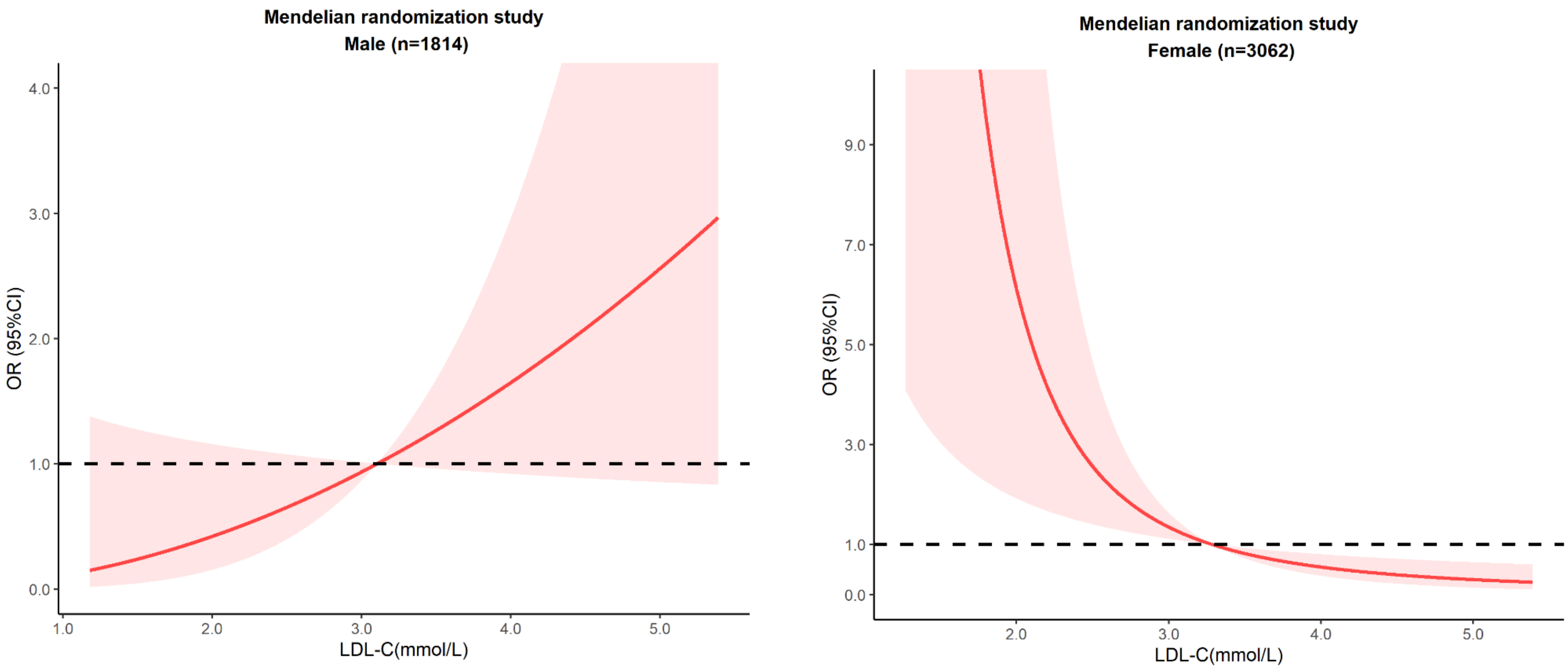

Supplementary figure 3 Nonlinear associations of LDL-C and diabetes in MR analyses stratified by sex

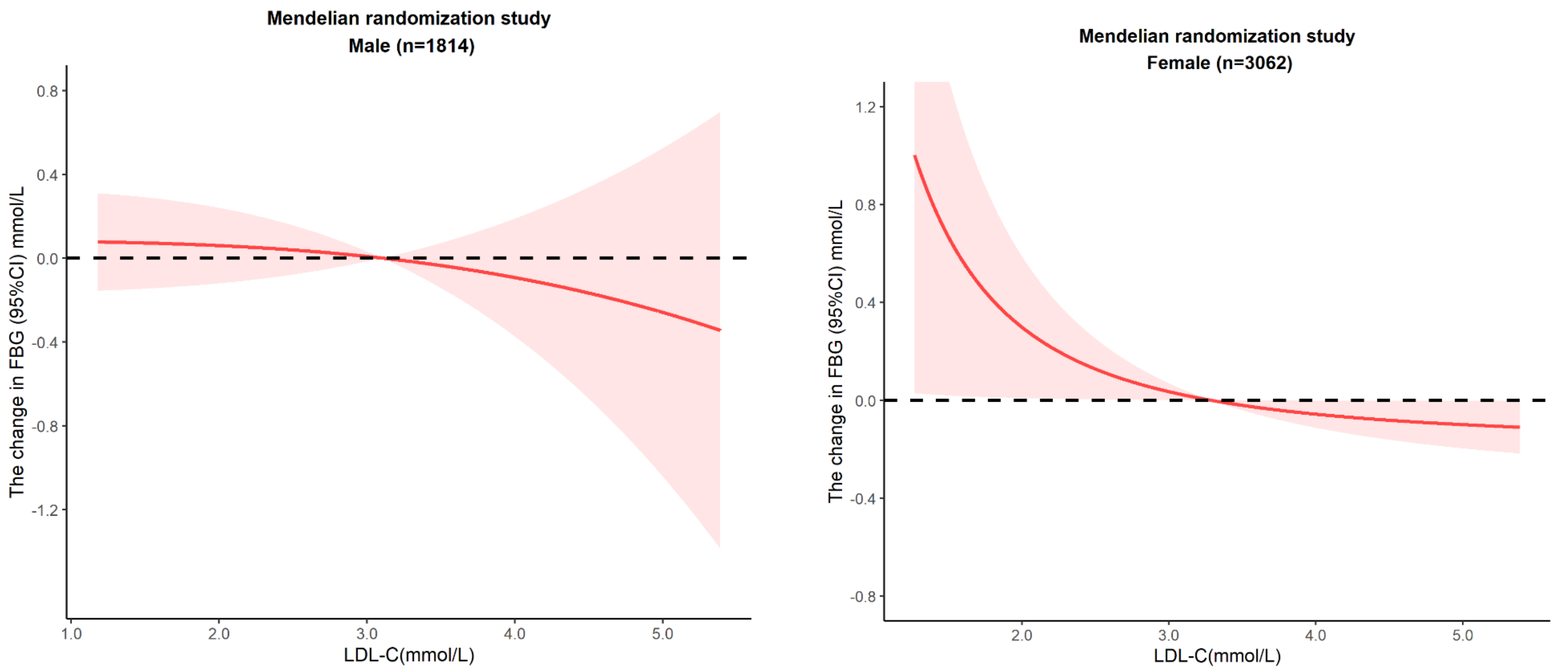

Supplementary figure 4 Nonlinear associations of LDL-C and FBG in MR analyses stratified by sex

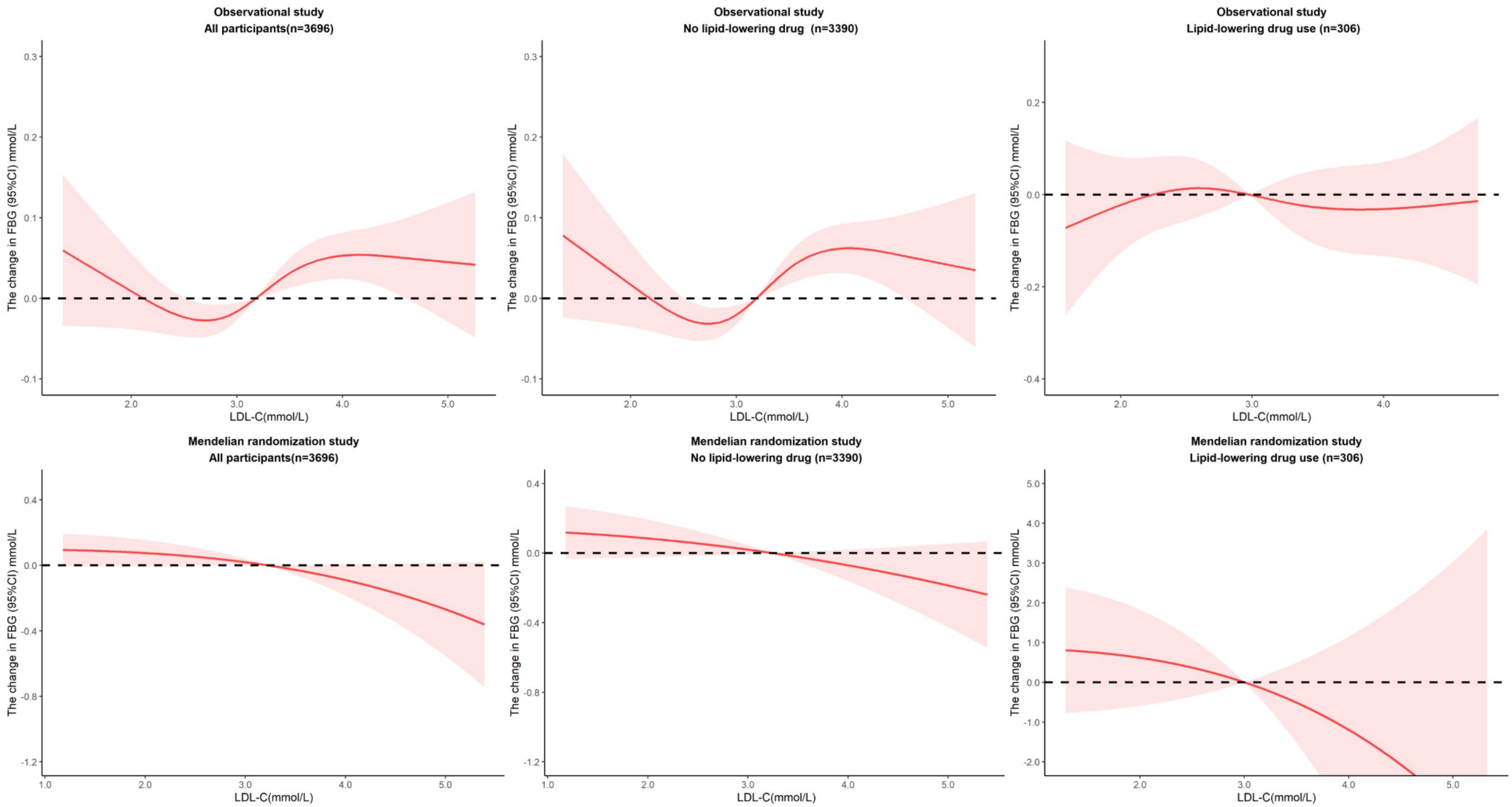

Supplementary figure 5 Nonlinear associations of LDL-C and FBG in observational analyses and MR analyses among participants without known diabetes or hypoglycemic drug use.
